# One AP2/ERF Transcription Factor Positively Regulates Pi Uptake and Drought Tolerance in Poplar

**DOI:** 10.3390/ijms23095241

**Published:** 2022-05-08

**Authors:** Ningning Chen, Jiajia Qin, Shaofei Tong, Weiwei Wang, Yuanzhong Jiang

**Affiliations:** Key Laboratory for Bio-Resources and Eco-Environment of Ministry of Education, College of Life Science, Sichuan University, Chengdu 610065, China; chenn130@foxmail.com (N.C.); qjj19980808@163.com (J.Q.); tongshaofei@foxmail.com (S.T.); wangww_infor@163.com (W.W.)

**Keywords:** *PalERF2*, transcriptional regulation, drought stress, inorganic phosphate starvation, *Populus*

## Abstract

Drought decreases the inorganic phosphate (Pi) supply of soil, resulting in Pi starvation of plants, but the molecular mechanism of how plants, especially the perennial trees, are tolerant to drought stress and Pi starvation, is still elusive. In this study, we identified an AP2/ERF transcription factor gene, *PalERF2*, from *Populus alba* var. *pyramidalis*, and it was induced by both mannitol treatment and Pi starvation. Overexpressing and knocking-down of *PalERF2* both enhanced and attenuated tolerance to drought stress and Pi deficiency compared to WT, respectively. Moreover, the overexpression of *PalERF2* up-regulated the expression levels of Pi starvation-induced (PSI) genes and increased Pi uptake under drought conditions; however, its *RNAi* poplar showed the opposite phenotypes. Subsequent analysis indicated that PalERF2 directly modulated expressions of drought-responsive genes *PalRD20* and *PalSAG113*, as well as PSI genes *PalPHL2* and *PalPHT1;4*, through binding to the DRE motifs on their promoters. These results clearly indicate that poplars can recruit *PalERF2* to increase the tolerance to drought and also elevate Pi uptake under drought stress.

## 1. Introduction

The inorganic phosphate (Pi) deficiency impairs plant growth and development [1]. This deficiency compels plants to evolve a series of morphological, physiological, and metabolic adaptations in order to improve Pi mobilization and uptake under low Pi circumstances, including increasing the activity of high-affinity Pi transporters, the induction of acid phosphatases (APases), and accumulation of anthocyanins [2,3]. In addition, the phosphorus cycling in woody plants may be different from herbaceous plants, because they experience seasonal change and the cycling growth may affect the dynamic changes of the total phosphorus content [4,5,6]. Drought stress leads to decreasing water uptake by roots, reducing leaf expansion and down-regulating stomatal conductance and causing a decrease in photosynthesis-mediated carbon assimilation [7,8]; it also decreases Pi supply through mineralization and by reducing Pi diffusion and mass flow in the soil [9,10,11]. This drought stress may reduce Pi uptake by influencing the nutrient uptake kinetics by roots [7,8,12] and therefore decrease Pi uptake from the soil and the concentration of phosphorus in plant tissue [13,14,15,16,17].

Pi starvation responses (PSR) of plants involve hundreds of Pi-starvation-induced (PSI) genes like *PURPLE ACID PHOSPHATASES* (*PAPs*), *PHOSPHATE TRANSPORTERS* (*PHTs*), *PHOSPHATE* (*PHO*), *PHOSPHATE RESPONSES* (*PHRs*), and *PHR-LIKES* (*PHLs*). Among them, PAPs can hydrolyze various phosphorus monoesters and release phosphorus under suitable pH conditions; PHTs can absorb phosphate from soil and redistribute Pi in plants [18,19,20,21,22]. Some PSI transcription factors also respond to dehydration. For instance, *PHRs* and *PHLs* belonging to the MYB-CC family have key and redundant functions in regulating plant transcriptional response to Pi starvation [23,24,25]; the ectopic expression of their homolog, *TaMYBsm3* of wheat, in *Arabidopsis* enhances drought tolerance [26]. AtWRKY75 is a positive regulator of Pi absorption through up-regulating the expression levels of *AtPHT1;1* and *AtPHT1;4* [27]; however, this gene has a negative function in osmotic tolerance [28]. *AtMYB2* involves salinity and drought responses [29,30,31] and is a transcriptional activator of the *miR399f*, which plays a crucial role in Pi homeostasis by repressing *PHO2* expression [32,33,34]. In poplars, there were more than 4000 and 9000 genes that showed differentiated expressions upon Pi starvation in roots and leaves under drought stress [35]. Moreover, the phosphate transporter (*PHT*) genes showed similar differentiated expressions upon drought stress [36,37]. Therefore, there is likely a crosstalk between PSR and dehydration responses.

The AP2/ERF superfamily is one of the biggest transcription factor families in plants [38]. The members of this family regulate target genes by binding to the GCC-box and some also can bind to dehydration-response element (DRE) motif [39,40]. The *AP2/ERF* genes are involved in various biotic and abiotic stress response, including wounding, pathogens, drought, and PSR. For example, AtORA59 integrates JA and ethylene signals to directly enhance the expression of *PDF1.2* and increase resistance against the fungus *Botrytis cinerea* in *Arabidopsis* [41]. *AtTINY* regulates brassinosteroid-mediated plant growth and drought responses [42], while *NtERF172* confers tobacco more drought tolerance by scavenging H_2_O_2_ [43]. In addition, around 22 *ERF* genes increase expressions in response to Pi starvation in *Jatropha curcas* [44], and the down-regulation of *ERF035* in this plant leads to changed root architecture and biosynthesis of anthocyanins under low Pi conditions [45]. In *Arabidopsis*, three *ERF* genes, *ERF1*, *ERF2* and *ERF5*, were suggested to be likely PHR1 targets [46].

In this study, we identified that *PalERF2*, an *AP2/ERF* gene from *P. alba* var. *pyramidalis*, was induced by drought stress and Pi starvation. Overexpression of *PalERF2* in poplars conferred more tolerance to drought stress and Pi deficiency, whereas knocking-down *PalERF2* by RNA interference (RNAi) attenuated tolerance to these two stresses. In addition, we found that the expression levels of PSI genes were up-regulated in the *PalERF2* overexpression lines, which resulted in an increase of Pi contents under drought condition, whereas the opposite phenotypes were observed in the *PalERF2* RNAi poplars. Moreover, *PalERF2* bound to the DRE motifs of the promoters of *PalRD20*, *PalSAG113*, *PalPHL2,* and *PalPHT1;4*, and, therefore, directly regulated their expressions. Therefore, these findings together suggest that *PalERF2* positively regulates the tolerance of poplar to Pi starvation and drought stress.

## 2. Results

### 2.1. Identification of a Drought and Low Pi Induced AP2/ERF Gene in P. alba var. pyramidalis

**Figure 1:**
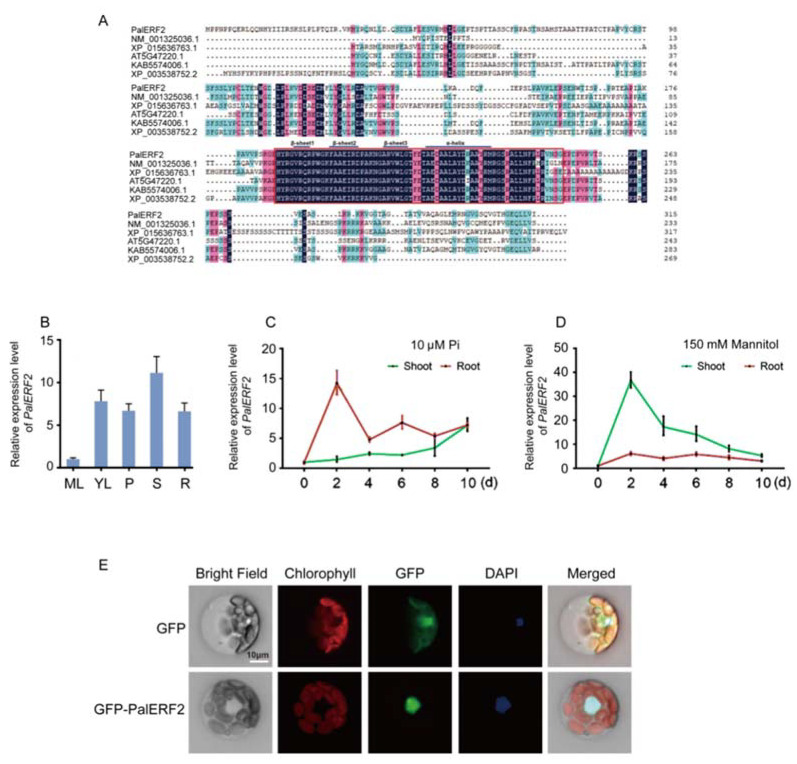
Characteristics of *PalERF2 of Populus alba* var. *Pyramidalis.* (**A**) Multiple sequence alignment of ERFs and the accession numbers are derived from different species (NM_001325036.1 from *Nicotiana tabacum*, XP_015636763.1 from rice, AT5G47220.1 from *Arabidopsis thaliana*, KAB5574006.1 from *Salix brachista,* and XP_003538752.2 from *Glycine max*). (**B**) The qRT-PCR analysis of *PalERF2* expression in root (R), stem (S), mature leaves (ML), young leaves (YL), and petiole (P) in the mediums of MS. (**C**,**D**) The temporal expression pattern of *PalERF2* under low Pi treatment (10 μM Pi) and 150 mM mannitol treatment in shoot and root, respectively. Error bars indicate SD values from three biological replicates. (**E**) Subcellular localization of PalERF2 in the mesophyll protoplasts of *P. alba* var. *pyramidalis*. The empty vector pBI221-expressing GFP is used as control (upper row) and the PalERF2-GFP fusion proteins are localized in the nucleus only (lower row). DAPI staining indicates the nucleus.

A 948bp length DNA fragment was simultaneously isolated from the cDNA pools of *P. alba* var. *pyramidalis* treated by both drought and low Pi, respectively. This transcript belongs to an AP2/ERF gene (PAYT003289.1), which is a homolog of *AtERF2* (*AT5G47220.1*) from *Arabidopsis.* Hence, we termed it as *PalERF2*. In *P. alba* var. *pyramidalis*, PalERF2 protein shares 71% sequence similarity with its closest paralog *PAYT035246.1*. PalERF2 is a member of the ERF subfamily B3 cluster [47] and contains a typical AP2 DNA-binding domain composed of an α-helix and three β-sheet regions (Figure 1A). Notably, PalERF2 and its homologs share the high identity only of the domain region (Figure 1A).

To determine the expression pattern of *PalERF2*, the expression level of *PalERF2* in various tissues of *P. alba* var. *pyramidalis* was examined by qRT-PCR. *PalERF2* expressed dominantly in the stem, and it had a similar level in young leaf, petiole, and root, but scarcely so in mature leaf (Figure 1B). In addition, we further determined the spatio-temporal expression pattern of *PalERF2*. Interestingly, both drought and low Pi induced *PalERF2* rapidly, and the maximum expression level was 2 days after treatments. However, low Pi treatment mainly induced *PalERF2* in root (Figure 1C), while drought-induced *PalERF2* was dominant in the shoot (Figure 1D). There are 23 ERF members of B3 cluster in poplar [47]. We chose the seven closest paralogs of *PalERF2* and analyzed their expression patterns under drought stress and low Pi condition (Figure S1A). These genes displayed various expression patterns, but no one was similar to *PalERF2* (Figure 1C,D and Figure S1B,C).

To verify the subcellular localization of PalERF2, we constructed a *35S:PalERF2-GFP* expression vector and transiently expressed it in mesophyll protoplasts of poplar. The results showed that the GFP protein as the control was distributed in both the cytoplasm and nucleus, while PalERF2 fused with GFP was only in the nucleus (Figure 1E).

### 2.2. PalERF2 Is a Positive Regulator of Poplar PSR

**Figure 2:**
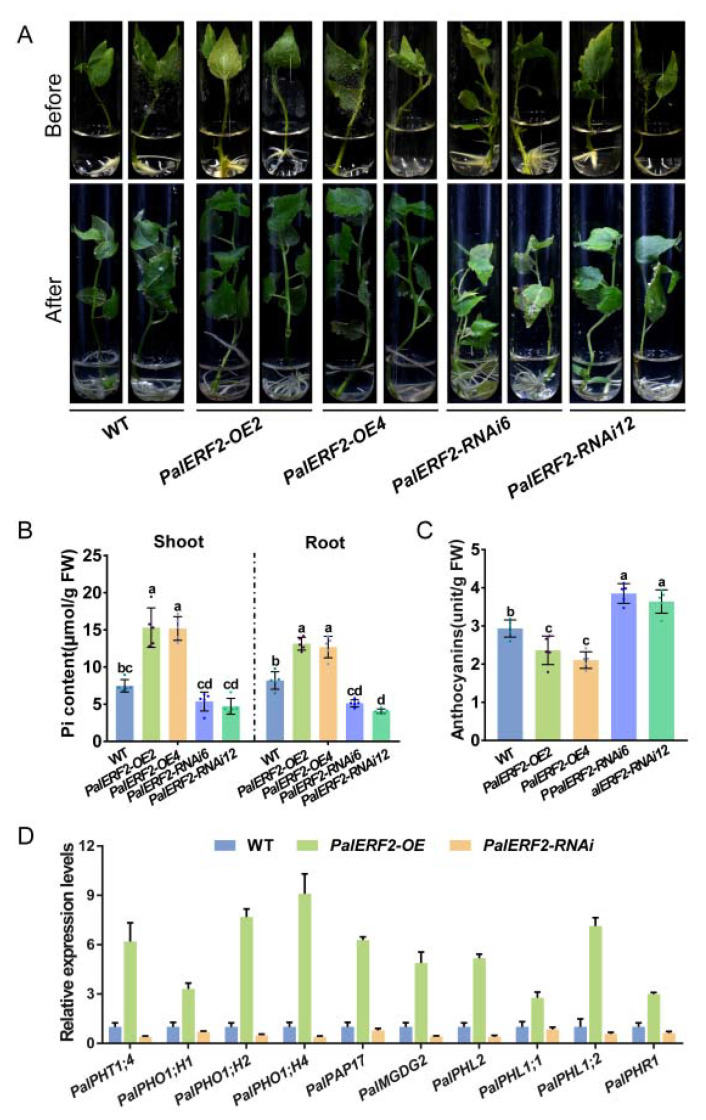
The *PalERF2* transgenic poplars under low Pi condition. (**A**) Phenotypes of transgenic and WT poplars grew in liquid medium with 10 μM Pi for 4 weeks. (**B**) The Pi contents of transgenic and WT poplars in root and shoot after low Pi treatment. (**C**) Anthocyanin contents of WT and transgenic poplar after low Pi treatment. Error bars indicate SD values from five biological replicates. Significant differences were analyzed by Duncan’s test (*p* < 0.05, *n* = 5). Different letters indicate statistically significant differences. (**D**) The qRT-PCR analysis of Pi starvation response (PSR) genes in *PalERF2-OE*, *PalERF2-RNAi,* and WT poplars. Error bars indicate SD values from three biological replicates.

To determine the function of *PalERF2* in tolerance to low Pi condition (10 μM Pi) in poplar, the overexpression lines (*PalERF2*-*OE2* and *PalERF2*-*OE4*) and RNAi-mediated gene knock-down lines (*PalERF2-RNAi6* and *PalERF2-RNAi12*) of *PalERF2* were obtained (Figure S2A–D), and the transgenic poplars show no significant difference in normal Pi condition (1.25 mM Pi) compared to the wild type (WT) (Figure S2C,D). However, after 4 weeks of growth in liquid MS containing 10 μM Pi, the *PalERF2-OE* transgenic poplars showed a stronger root system and higher shoot, but the *PalERF2-RNAi* lines showed attenuated growth in the plant height, leaf, and root system (Figure 2A and Figure S3). Moreover, the Pi contents in the root and shoot of the *PalERF2-OE* lines were obviously highest, and the *PalERF2-RNAi* cuttings had the lowest Pi contents in the whole plants. The anthocyanin accumulation is an indicator of low Pi stress degree. Compared to WT, the overexpression of *PalERF2* significantly reduced anthocyanin accumulation under Pi starvation, but *PalERF2-RNAi* poplars accumulated the most anthocyanins (Figure 2B,C). These results indicated that *PalERF2* is a positive regulator involved in tolerance to Pi starvation in poplar.

To investigate whether overexpression or knocking-down of *PalERF2* affects the expression of PSI genes, we analyzed the expression level of these genes in the transgenic and WT plants by qRT-PCR. As shown in Figure 2D, *PalPHT1;4*, a Pi transporter [20], was up-regulated in overexpression lines. *PHL1*, *PHL2,* and *PHR1* are considered to be key and function-redundant transcription factors in response to Pi starvation in plants [25], and *PalPHL1;1*, *PalPHL1;2*, *PalPHR1,* and *PalPHL2* were strongly upregulated in *PalERF2-OE* poplars. In addition, the *PHO1s* [48], such as *PalPHO1;H1*, *PalPHO1;H2,* and *PalPHO1;H4*, which are responsible for transferring Pi to the xylem and ultimately into the stem, were also up-regulated in transgenic plants. However, the above PSI genes are down-regulated in *PalERF2-RNAi* poplars. *SPX3,* which encoded a repressor of PSR by interacting with OsPHR2 in rice [49], was up-regulated in overexpressing *PalERF2* poplars and down-regulated in *PalERF2*-RNAi lines. A purple acid phosphatase gene *PalPAP17* [50] and a type B monogalactosyldiacylglycerol synthase gene *PalMGDG2* [51], showed opposite expression patterns in overexpressing and knocking-down poplars. These results indicated that *PalERF2* directly or indirectly regulate some PSI genes.

### 2.3. PalERF2 Directly Regulated Expression of PalPHT1;4 and PalPHL2 through Binding to the DRE Element in Their Promoters

ERF subfamily members can bind to GCC box (5′ AGCCGCC 3′) or dehydration-responsive element (DRE) (5′ A/GCCGAC 3′) [39,40,52,53]. We analyzed the promoters of PSI genes whose expression had been up-regulated in the *PalERF2* overexpressing poplars, however, there was no GCC box in these promoters. We found that some of these genes contained DRE or core DRE sequence on their promoters (Figure S4). For example, one and two DRE elements were found in the *PalPHR1* and *PalMGDG2* promoters, respectively. All promoters of *PalPHT1;4*, *PalPHL2,* and *PalMGDG2* contain a core DRE sequence (5′ CCGAC 3′).

**Figure 3:**
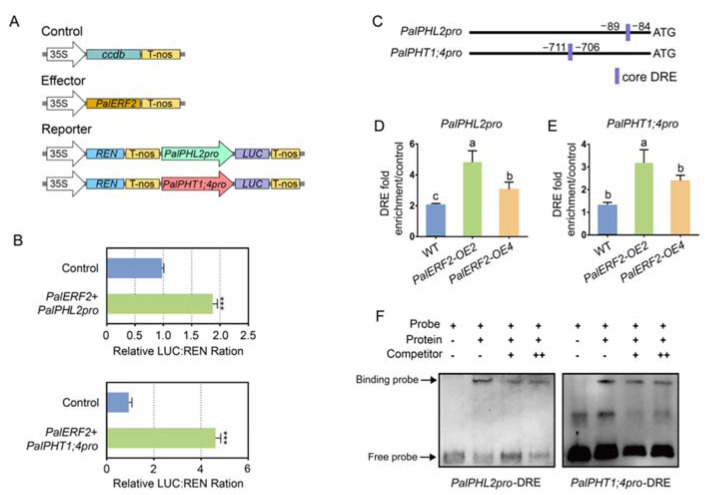
PalERF2 regulates *PalPHL2* and *PalPHT1;4* expression. (**A**) Structures of effector and reporters employed in dual-luciferase assay. (**B**) Transient co-expression of effector and reporter vectors in *Nicotiana benthamiana* leaves for dual-luciferase assay. Error bars indicate SD values (*n* = 3). Asterisks indicate significant differences compared to control by Student’s *t*-test, ***, *p* < 0.01. (**C**) Distribution of core DRE motifs in the promoters of *PalPHL2* and *PalPHT1;4*. (**D**,**E**) ChIP-qPCR determined the binding of PalERF2 to the *PalPHL2* and *PalPHT1;4* promoter regions containing DRE, respectively. Error values represent means ± SD (*n* = 3). Significant differences were analyzed by Duncan’s test (*p* < 0.05, *n* = 5). Different letters indicate statistically significant differences. (**F**) EMSA tested the binding activity of PalERF2 to the DRE in *PalPHL2* and *PalPHT1;4* promoters. The unlabeled cold probes were added to compete with labeled probes. + means the cold probe is 20 times the labeled probe, ++ means 50 times. The arrows mark the binding probe and free probe.

To confirm whether PalERF2 could bind to the DRE elements of these promoters, we chose *PalPHT1;4* and *PalPHL2* for further confirmation. We determined again that PalERF2 could significantly up-regulate the transcription of their promoters by a dual-luciferase assay (Figure 3A,B). Further ChIP-qPCR indicated that PalERF2 could bind to the promoter regions, harboring DRE elements of the *PalPHT1;4* and *PalPHL2* in vivo (Figure 3C,D). Subsequent EMSA indicated that PalERF2 bound to DRE element of *PalPHT1;4* and *PalPHL2* in vitro; such a binding could be impaired by competitors (Figure 3F). Together, these findings suggest that PalERF2 directly and positively regulated *PalPHT1;4* and *PalPHL2*.

### 2.4. PalERF2 Positively Regulates Drought Stress of Poplar Cuttings

**Figure 4:**
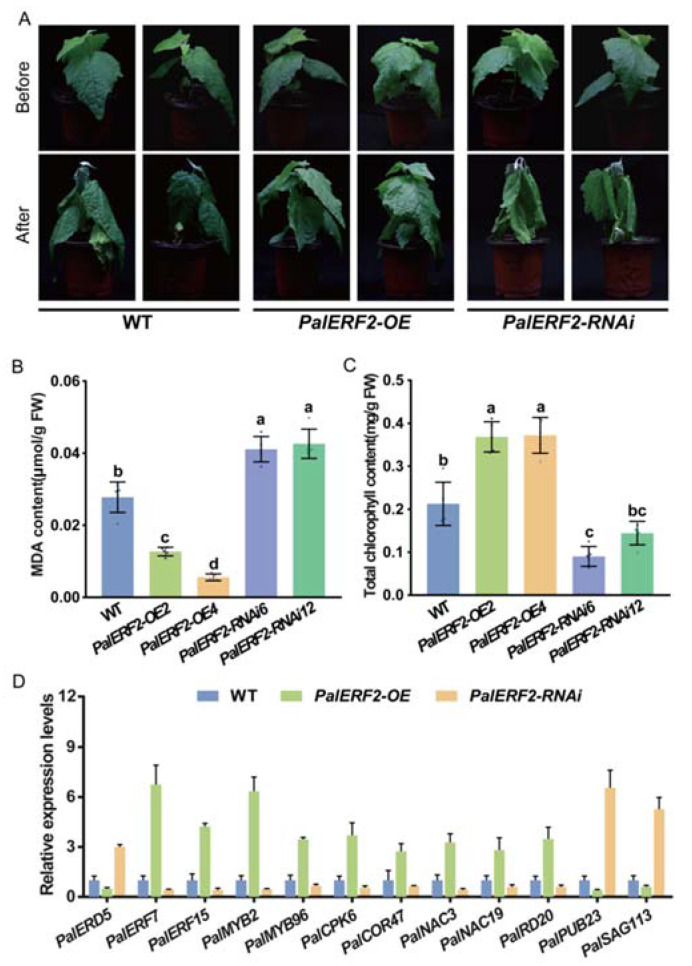
The phenotypes of *PalERF2* transgenic poplars under drought stress. (**A**) The phenotypes of transgenic and WT poplars after 5 days of drought treatment. (**B**) The MDA contents were measured after drought treatment. (**C**) The total chlorophyll contents were measured after drought treatment. (**B**,**C**) values represents means ± SD (*n* = 5). Significance of differences was analyzed by Duncan’s test (*p* < 0.05, *n* = 5). Different letters indicate statistically significant difference. (**D**) The relative expression of drought-associated genes in *PalERF2-OE*, *PalERF2-RNAi,* and WT poplars. Error bars indicate SD values from three biological replicates.

To determine the function of *PalERF2* in poplar tolerant to drought stress, the cuttings of *PalERF2* overexpressing and knocking-down lines were transplanted to and cultivated in the soil. After 3 weeks, these plantlets were withdrawing water for drought treatment. After 5 days of drought treatment, the leaves of *PalERF2-RNAi* poplars showed more severe dehydration than WT, whereas the leaves of overexpressing poplars had slightly dropped (Figure 4A). Moreover, RNAi plants contained the highest levels of MDA and the least total chlorophyll among three genotypic poplars, in contrast, *PalERF2-OE* lines had the lowest MDA contents and the highest total chlorophyll contents (Figure 4B,C). These results indicate that the RNAi poplars were most stressed and overexpression lines were most tolerant. Therefore, *PalERF2* had a positive function in tolerance of poplars to drought stress.

To reveal the influence of *PalERF2* on the expression of drought stress-related genes, the qRT-PCR was used for detecting the expression differences of drought stress-related genes in *PalERF2* transgenic lines and WT plants. As shown in Figure 4D, the expression level of *PalERD5* was down-regulated in *PalERF2-OE2* and *PalERF-OE4* but up-regulated in *PalERF2-RNAi* lines compared with WT. Its homolog *AtERD5* encodes a mitochondrial proline dehydrogenase and its transcription is repressed by dehydration in *Arabidopsis* [54]. In addition, four transcription factor genes, *PalMYB2, PalMYB96, PalNAC3,* and *PalNAC19*, whose homologs show positive responses to drought and ABA signaling in other plants [55,56,57,58], were also expressed higher in overexpressing plants and lower in RNAi lines compared to WT. *PalSAG113* was down-regulated in *PalERF2-OE* plants and its homolog was found to be a negative regulator of ABA signaling [59]. We also found that a likely gene with the E3 ubiquitin ligase homolog [60], *PalPUB23,* also decreased expression levels in overexpressing cuttings and up-regulated expression in RNAi poplars. In addition, the expression of *PalCPK6* and *PalCOR47*, whose homologs respond to drought stress [61,62,63], were also up-regulated in overexpression lines and down-regulated in the RNAi lines respectively. We found that the expression of *PalRD20* was significantly enhanced in overexpression lines but decreased in the RNAi lines. The homolog of this gene, *AtRD20,* is a stress-inducible caleosin and participates in drought tolerance in *Arabidopsis* [64]. These results indicate that PalERF2 up-regulated the expression of the drought-responsive genes and down-regulated the genes that negatively modulate drought response.

### 2.5. PalERF2 Regulated Expression of PalRD20 and PalSAG113 through Binding to the DRE Motif of Its Promoter

**Figure 5:**
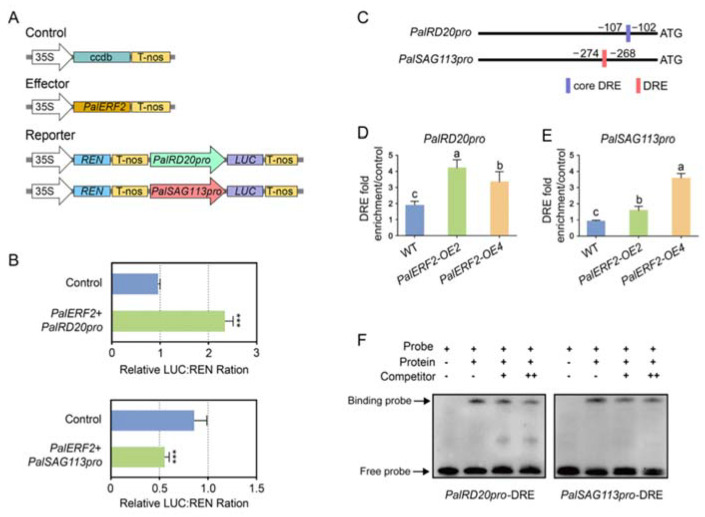
PalERF2 directly regulated the expression of *PalRD20* and *PalSAG113*. (**A**) Structures of effector and reporters employed in Dual-luciferase assay. (**B**) Transient co-expression of effector and reporter vectors in *N. benthamiana* leaves. Data shown as mean ± SD (*n* = 3). Asterisks indicate significant differences compared to control by Student’s *t*-test, ***, *p* < 0.01. (**C**) Distribution of DRE and core DRE motifs in the promoter of *PalRD20* and *PalSAG113*. (**D**,**E**) ChIP-qPCR demonstrated that PalERF2 bound to the promoter region of *PalRD20* and *PalSAG113* containing DRE in vivo. Significant differences were analyzed by Duncan’s test (*p* < 0.05, *n* = 5). Different letters indicate statistically significant differences. (**F**) EMSA demonstrated that PalERF2 bound to the DRE in the *PalRD20* and *PalSAG113* promoters. Unlabeled cold probes as a competitor to compete with labeled probes. + means the cold probe is 20 times the labeled probe, ++ means 50 times. The arrows mark the binding probe and free probe.

To investigate whether PalERF2 directly regulated these drought-related genes, we analyzed promoters of the genes with differential expressions in *PalERF2* transgenic lines compared to WT. We found that there was no GCC box, but at least one DRE element or one core DRE motif in promoters of these genes (Figure S5). For instance, the promoters of *PalCOR47*, *PalPUB23,* and *PalSAG113* had one DRE element, respectively, while the *PalRD20* and *PalNAC19* promoters contained a core DRE motif, respectively. We hypothesized that PalERF2 could directly regulate the genes containing DRE elements in the promoters. We firstly determined PalERF2 could increase the transcription activity of *PalRD20* and *PalSAG113* promoters by a dual-luciferase assay. The results showed that PalERF2 significantly enhanced the fluorescence intensity of *PalRD20* promoter driven by *LUC* compared to the control. However, it repressed the expression of *LUC* driven by *PalSAG113* promoter (Figure 5A,B). ChIP-qPCR assay showed that PalERF2 binds to the promoter regions containing DRE elements in *PalRD20* and *PalSAG113* overexpression lines in vivo (Figure 5C,D). The EMSA assay showed that the DRE element in the promoters of *PalRD20* and *PalSAG113* could be bound by MBP-PalERF2 fusion protein, and this binding could be taken apart by cold probes in vitro (Figure 5F). Therefore, PalERF2 directly regulates the expression of *PalRD20* and *PalSAG113* through the DRE element in their promoters.

### 2.6. Overexpressing PalERF2 Improved Pi Uptake of Poplars and Expression Level of PSI Genes during Drought Stress

**Figure 6:**
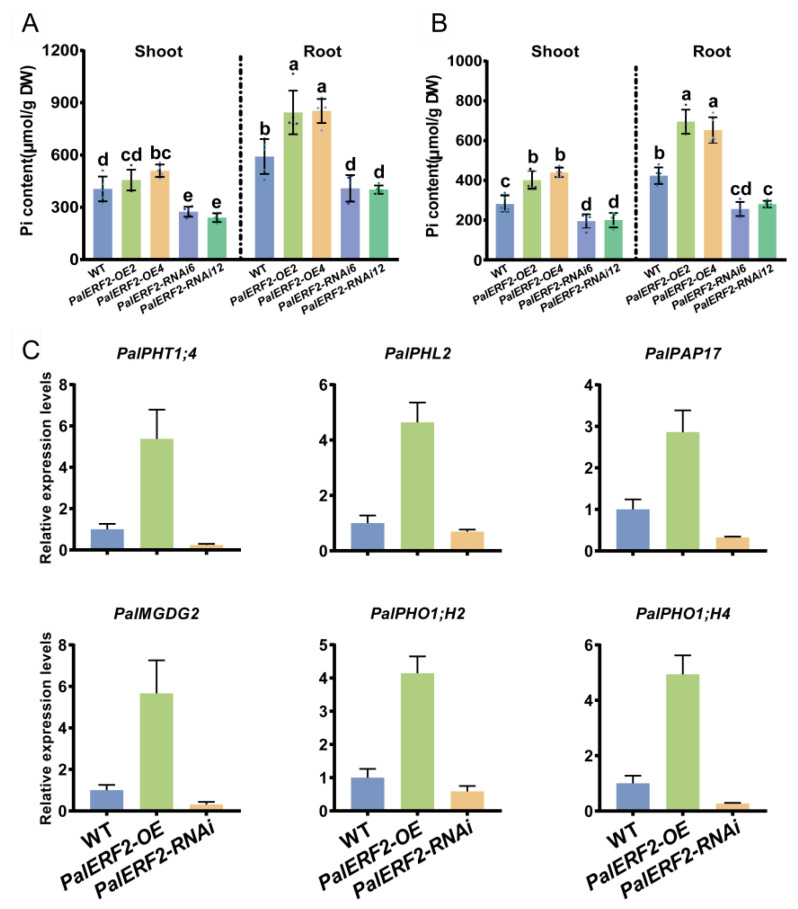
The Pi contents and the expression of PSR genes in *PalERF2* transgenic and WT poplars. (**A**) The Pi contents in WT and transgenic plants before drought treatment. (**B**) The Pi contents in WT and transgenic poplars after drought treatment. (**A**,**B**) Error bars indicate SD values from three biological replicates. Significant difference was analyzed by Duncan’s test (*p* < 0.05, *n* = 5). Different letters indicate statistically significant differences. (**C**) The qRT-PCR analyzed the relative expression of PSR genes in WT and transgenic poplars after drought treatment. Error bars indicate SD values from three biological replicates.

Because drought stress leads to reducing Pi diffusion and mass flow in the soil, we wondered whether PalERF2 could increase the tolerance to Pi starvation that resulted from drought stress. Therefore, we measured the Pi contents of *PalERF2* transgenic poplars before and after drought treatment. Before the drought treatment, overexpressing plants had more abundant Pi contents both in the shoot and root compared to WT, whereas the Pi contents of RNAi lines were lower than that of WT (Figure 6A). Although drought stress decreased Pi contents in all lines and tissues, the *PalERF2* overexpression lines contained much more and knocking-down lines had less Pi contents compared to the WT (Figure 6B). In addition, we used qRT-PCR to detect the expression levels of PSI genes after drought treatment, and these genes showed significantly enhanced expression levels in the overexpression lines and decreased expression levels in RNAi lines compared to the WT (Figure 6C). These results indicated that drought stress impairs the Pi uptake capacity of poplar, but up-regulated expression of *PalERF2* can rescue Pi absorption when drought stress occurs.

## 3. Discussion

The occurrence of one abiotic stress is usually accompanied by several secondary stresses in plants. For example, water deficiency not only leads to osmotic stress but also reduces Pi uptake in plants [13,14,15,16,17]. The responses of plants to drought stress involve the transcriptional rearrangements of associated genes including a series of PSI genes [65]. For example, the micro-RNA, *miR399f* modulates plants response to drought, ABA, and salt stresses, and also plays a crucial role in Pi homeostasis by repressing *PHO2* expression in *Arabidopsis* [32,33,34]. The transcriptional activator of the *miR399f*, AtMYB2 is involved in salinity and drought response [29,30,31]. Therefore, the enhancement of Pi uptake is one of the strategies for plants to adapt to drought-caused Pi starvation. Herein, we revealed that *PalERF2* from *P. alba* var. *pyramidalis* was induced by mannitol treatment and low Pi condition (Figure 1C,D) and demonstrated it was a positive regulator of tolerance to drought and Pi starvation in poplar (Figure 2 and Figure 4).

Stomatal closure indicates a response to drought stress in plants, and thus many genes regulating stomatal movements change the expression in leaf under drought stress [66]. Mannitol treatment simulates drought stress but it does not affect Pi diffusion and mass flow. Our mannitol treatment rapidly induced *PalERF2* expression in the shoot of poplar, and overexpression of *PalERF2* resulted in more tolerance to drought stress compared to the WT plants (Figure 1D and Figure 4A). PalERF2 directly up-regulated the expression of *PalRD20* (Figure 5). In *Arabidopsis*, *RD20* is mainly expressed in leaves, guard cells. and flowers, and positively regulates stomatal closure [64]. This also strengthened ABA signaling through decreasing the transcription of *PalSAG113* (Figure 5), a repressor of the ABA pathway [67]. Therefore, *PalERF2* was induced rapidly in the shoot in order to close stomata under drought conditions. In addition, the root system is mainly responsible for Pi uptake; thereby, the associated genes prefer to express in the root. For example, a total of 42 *PHT* genes were identified in another *P. trichocarpa*, of which 25 *PHTs* were highly expressed in roots [37]. After Pi starvation treatment, *PalERF2* was mainly induced in poplar roots (Figure 1C), and PalERF2 directly and positively regulated the expression of two PSI genes, *PalPHL2* and *PalPHT1;4*, to improve the Pi uptake in poplar (Figure 2 and Figure 3). In *Arabidopsis*, AtPHL2 functions redundantly with AtPHR1 to control transcriptional responses to Pi starvation and can directly bind to the P1BS elements of *PHT1s* promoters to regulate expression [25]; the homolog of *PalPHT1;4* in *Arabidopsis*, *AtPHT1;4,* is the main high-affinity Pi transporter in roots [20]. Therefore, the overexpression of *PalERF2* improved the Pi uptake of poplars and enhanced the growth in the Pi starvation environment. These results suggest that the double functions of *PalERF2* rely on its induced expressions in specific tissues. Although *PalERF2* expression was mainly induced in shoots after 150 mM Mannitol treatment, it was also upregulated in roots (Figure 1D). This implies that *PalERF2* can enhance Pi uptake in poplar to some extent under drought condition. Our results of the Pi contents determination of PalERF2 transgenic poplars before and after drought treatment support this conclusion, and some PSI genes like *PalPHT1;4* and *PalPHL2* showed significantly enhanced expression in *PalERF2-OE* poplars but decreased expression in the *PalERF2-RNAi* lines after drought treatment (Figure 6). These results indicate that PalERF2 participates in the drought and Pi starvation stress responses of poplar through tissue-specific transcription networks, but at the same time can enhance the Pi uptake of poplar under drought stress.

DRE is bound by the DREB proteins, such as *DREB1, DREB2,* and *CBF1*, which belong to a subfamily of the AP2/ERF family [40]. In addition, another subfamily of AP2/ERF members, ERFs, can bind to DRE and GCC box. For instance, AtERF1, AtERF4, and AtEBP exhibit similar binding activities to the DRE and GCC boxes in *Arabidopsis* [54]. AtERF1B binds to the DRE of *RD29B*, *RD20,* and *ERD7* promoters to regulate the expressions of these genes under drought and salinity stress [68]. PalERF2 is a member of the ERF subfamily, and overexpressing or knocking-down this gene thus influences the expression levels of drought-responsive gene *PalRD20* through the DRE element but not GCC box (Figure 5). PalERF2 targets PSI genes, like *PalPHL2* and *PalPHT1;4,* also through the DRE box (Figure 3). Therefore, the PalERF2 modulates target genes depending on the DRE element. Although DRE is an element of the promoters of many ABA-independent drought-responsive genes [68,69,70], overexpressing or knocking-down of *PalERF2* therefore also regulated the expression level of ABA-dependent drought-responsive genes, including *PalNAC19*, *PalNAC3*, *PalMYB96,* and *PalSAG113* (Figure 4D). Moreover, PalSAG113 is a negative regulator of the ABA pathway [64]. These results indicate that *PalERF2* may orchestrate ABA-dependent and -independent pathways. Remarkably, *PalERF2* displays bifunction to the target genes, because it activates the transcription of *PalRD20*, *PalPHL2,* and *PalPHT1;4*, but represses the expression of *PalSAG113* (Figure 5B). This suggests that PalERF2 may combine with other transcription regulatory proteins to modulate the transcription of all of these target genes.

**Figure 7:**
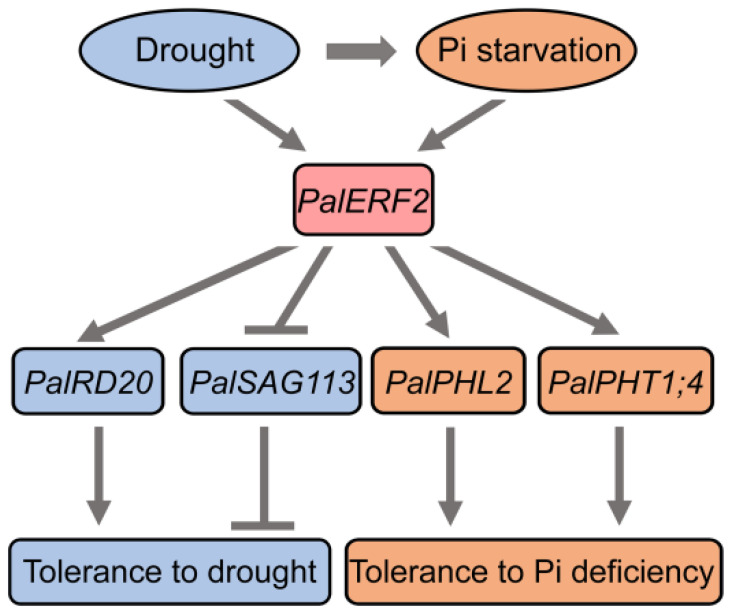
The proposed model for the PalERF2 mediated drought stress and low Pi responses in poplars.

Our results together indicate a model for *PalERF2* to mediate PSI genes and increase tolerance to drought stress in poplar (Figure 7). When drought stress and Pi starvation occur, PalERF2 is induced. PalERF2 is recruited to up-regulate the transcription of *PalRD20* and down-regulate *PalSAG113* expression, resulting in enhanced tolerance to drought. In addition, PalERF2 also positively regulates the expression level of *PalPHL2* and *PalPHT1;4* to increase Pi uptake, hence increasing tolerance to Pi deficiency. Our results therefore provide new insights into molecular crosstalk between drought and Pi starvation in woody plants.

## 4. Materials and Methods

### 4.1. Plant Materials and Growth Conditions

The plantlets of *P. alba* var. *pyramidalis* and *P. tomentosa* were propagated in woody plant medium (WPM, Hopebio, Qindao, China) with 30 g·L^−1^ sucrose and 500 μL·L^−1^ PPM (Plant Cell Technology, Washington, DC, USA). The growth chamber provided a 16 h of light/8 h of dark cycle and 100 μmol·m^−2^·s^−1^ light intensity at a constant temperature of 25 °C. For the Pi starvation treatment, poplar shoots of the same length were selected for rooting culture in WPM medium containing 0.1 mg/L NAA until each line took root, and then they were transferred to sterile tubes with 5 mL MS Pi-deficient liquid medium and went on growing for 4 weeks. In the MS Pi-deficient medium, KH_2_PO_4_ was replaced by equimolar amounts of KCl_2_ and another 10 μM KH_2_PO_4 was_ added. For the detected expression levels of *AP2*/*ERF* genes, *P. alba* var. *pyramidalis* were cultured in 5 mL MS Pi-deficient (10 μM) liquid medium or MS liquid medium with 150 mM mannitol, then shoots and roots were collected every other day, respectively. RNA from shoots and roots were extracted and reverse transcription performed. In order to perform drought treatment, the poplar plantlets with similar growth status and scale were transferred to the soil for 3 weeks, then watering was cut off until the phenotype appeared.

### 4.2. Nucleic Acid Extraction and qRT-PCR Analysis

The genome DNA (gDNA) of poplar were extracted by CTAB method [71]. Total RNA from poplar were extracted by Biopin Plant Total RNA Extraction Kit (Bioflux, Beijing, China) and gDNA was removed by RNAase-free DNase I (TaKaRa, Dalian, China). Following, reverse transcription of 2 μg of RNA was carried out to obtain complementary DNA (cDNA) using a PrimeScriptTM RT Reagent Kit (Takara, Dalian, China). The quantitative RT-PCR assay was performed with Real Time PCR East^TM^-SYBR Green II (Foregene, Chengdu, China), and ubiquitin (*UBQ*) gene was used as an internal reference. All gene-specific primers are listed in Table S1.

### 4.3. Gene Cloning

The coding sequence (CDS) of *PalERF2* was obtained by PCR, and the parameters are as follows: 95 °C initial denaturation for 5 min, 34 cycles of 95 °C for 30 s, 55 °C for 30 s and 72 °C for 30 s, and final extension at 72 °C for 5 min. The Phanta Max Super-Fidelity DNA polymerase (Vazyme, Nanjing, China) was used for the PCR reaction. The CDS of *PalERF2* was ligated onto the pCX-DG vector by Seamless Cloning Mix Kit (Biomed, Beijing, China) and the construct was introduced into the *Agrobacterium* strain GV3101 by freeze-thaw method [72].

### 4.4. Generation of Transgenic Poplars

*PalERF2* transgenic poplars (overexpression and RNAi) obtained by *Agrobacterium* mediated the leaf discs transformation method [73]. The key points of the method are as follows: The transgenic *Agrobacterium* was cultured to OD_600_ 0.4–0.6, then centrifuged to remove the supernatant and resuspended in WPM liquid medium containing 100 μmol/L acetosyringone (AS); healthy poplar leaves were selected and cut along the main leaf vein to grow 1.5 cm square and placed in the resuspended *Agrobacterium* for 10 min; the excess bacteria on the leaves were removed and placed on solid WPM containing 100 μmol/L AS for co-cultivation for 2 days. After selective cultivation, budding, and rooting cultivation, a complete poplar tree was finally obtained. The positive transformants were determined by PCR and the overexpression lines with the highest expression levels and RNAi lines with the lowest expression levels were analyzed by qRT-PCR. The primers are shown in the Table S1.

### 4.5. Subcellular Localization of PalERF2

*PalERF2* CDS fragment was ligated onto the pBI221 vector. Then the recombinant vector was introduced into poplar mesophyll protoplasts and the cell nucleus was stained by 4’,6-diamidino-2-phenylindole (DAPI). The protocol isolated poplar mesophyll protoplasts according to the previous description [74]. Healthy poplar leaves were selected and cut into 0.2 mm diameter filaments, and placed in 50 mL enzymatic hydrolysis solution containing 0.75 g Cellulase R10 and 0.2 g Macerozyme R10 (YAKULT, Kyoto, Japan). Enzymatic digestion was carried out in the dark for 3 h. Then, a 50 mL W5 solution (154 mM NaCl, 125 mM CaCl_2_, 5 mM KCl and 2 mM MES) was added to stop the enzymatic hydrolysis, and the protoplasts were collected by centrifugation at 100× *g*. MMG solution (0.4 M Mannitol, 15 mM MaCl_2_ and 4 mM MES) was used to resuspend the protoplasts, 200 μL protoplast/MMG solution was taken, a 1 μg vector was added, and then 220 μL of 40% PEG solution was added to the mix; 800 μL of W5 solution was added and 100 g centrifugation was carried out to collect the protoplasts; then protoplasts were resuspended in 1 mL W5 solution and cultured for 10 h at 22 °C under low light. Green fluorescence was observed by confocal laser microscope (Leica TCS SP5 II system, Solms, Germany).

### 4.6. Dual-Luciferase Assay

The promoters of *PalPHL2*, *PalPHT1;4*, *PalRD20,* and *PalSAG113* were obtained by PCR using sequence-specific primers, and the PCR products were ligated onto pGreen II 0800-LUC vector as the reporters using a Seamless Cloning Mix Kit (Biomed, Beijing, China). The construct pCX-DG-*PalERF2* was set as the effector. All vectors were introduced into *Agrobacterium* strain GV3101 by freeze-thaw method. The *Agrobacterium* was cultured in YEP medium to OD_600_ 0.6–0.8, and the cells were collected by centrifugation at 5000× *g* and resuspended in an infection buffer (10 mM MgCl_2_, 10 mM MES, and 100 μmol/L AS, pH 5.7), cultured at 200 rpm at 28 °C for 2 h. Then the reporter and effector were co-injected into leaves of *Nicotiana benthamiana.* After 2 days of dark treatment and 1 day of normal growth, the LUC and REN luciferase signals were detected by Dual-luciferase Reporter System (Synergy H1, BioTek, Winooski, VT, USA) using a Luciferase Reporter Assay Kit (Biovision, San Francisco, CA, USA).

### 4.7. Measurement of Anthocyanin Content

The weighed leaves of the WT and transgenic poplars were homogenized with 1 mL hydrochloric acid/methanol (*v*/*v*, 1/99) to extract anthocyanin at 4 °C until the leaves turned white. The values of OD_530_ and OD_657_ for each sample were measured by a spectrophotometer (AOE, Shanghai, China). The anthocyanin calculation formula is (A_530_ − 0.25·A_657_)/fresh weight [75].

### 4.8. Measurement of Phosphate Content

The phosphorus content was measured as described previously with some modifications [76]. The weighed fresh or dry poplar root and shoot were shattered with a high-throughput grinder (SCIENTZ-48, Ningbo, China) and mixed with 100 μL of phosphorus extract buffer (0.2922 g of EDTA, 1.21 g of Tris, 5.844 g of NaCl, 700 μL β-mercaptoethanol, and 100 mM PMSF constant volume to 1 L by ddH_2_O) and 900 μL 1% acetic acid; then they were incubated at 42 °C for 30 min. After centrifugating the suspension at 12,000× *g* for 5 min, 150 μL of the supernatant was transferred into a new tube with 350 μL color-developing solution (0.35 g ammonium molybdate, 2.339 mL concentrated sulfuric acid, and 1.4 g ascorbic acid constant volume to 100 mL by ddH_2_O) and incubated at 42 °C for 30 min. Finally, the absorbance at the wavelength of 820 nm was determined, and the calculation of the phosphorus content was according to the standard curve.

### 4.9. Measurement of MDA and Total Chlorophyll Content

For the MDA content measurement, The weighed leaves of WT and transgenic poplars were homogenized with 1 mL 5% trichloroacetic acid (TCA) by a high-throughput grinder (SCIENTZ-48, Ningbo, China). After centrifugation at 3000× *g* for 10 min, 200 μL of the supernatant was mixed with an equal volume of 0.67% thiobarbituric acid (TBA). Then it was incubated at 100 °C for 30 min, and centrifuged again to remove the precipitate, and the supernatant was measured with absorbances at 450 nm, 532 nm, and 600 nm by an ultraviolet spectrophotometer (AOE, Shanghai, China), respectively. The MDA calculation formula is [6.45·(A_532_ − A_600_) − 0.56·A_450_]/fresh weight [77]. 

For the total chlorophyll content measurement, 1 mL of 80% acetone was used to extracted chlorophyll until the leaves turned white, then the absorbance was measured at 663 nm and 645 nm, respectively. The chlorophyll content calculation formula is (8.02·A_663_ + 20.21·A_645_)/(1000*fresh weight) [78].

### 4.10. Electrophoresis Mobility Shift Assay (EMSA)

The CDS of *PalERF2* was ligated onto the pMAL-c2x vector and introduced into the *Escherichia coli* strain Rosetta. Positive transformants were cultured at 37 °C until OD_600_ reached 0.6, then 1% IPTG (*m*/*v*) was added for 16 h at 16 °C. The MBP-PalERF2 fusion protein was purified by Amylose Resin (NEB Inc., Ipswich, MA, USA). Then, 45 bp-length probes containing a DRE element from promoters of *PalPHL2*, *PalPHT1;4*, *PalRD20*, and *PalSAG113* were labelled by biotin. The EMSA was according to the protocol of the LightShift^®®^ Chemiluminescent EMSA Kit (Thermo Scientific, Waltham, MA, USA). The probes and primers are listed in Table S1.

### 4.11. Chromatin Immunoprecipitation-qPCR (ChIP-qPCR)

Transgenic poplar of *PalERF2* tagged with GFP were transplanted into nutritional soil for 1 month, and then 3 g fresh leaves were used for CHIP-qPCR assay according to the previous description [79]. After formaldehyde cross-linking, nucleoprotein extraction, sonication of DNA, addition of Anti-GFP, protein A beads binding protein, protein digestion, DNA extraction, and other steps, the DNA was finally obtained, and qPCR was used to detect whether the specific DNA fragment was enriched. The primers are listed in Table S1.

### 4.12. Phylogenetic Analysis

The sequence data of AP2/ERF genes were downloaded from the NCBI database (www.ncbi.nlm.nih.gov, accessed on 17 April 2020). The amino acid sequences were aligned and generated a phylogenetic tree using Neighbor-Joining (NJ) method by the MEGA6 software. The bootstrap value was 1000.

### 4.13. Statistical Method

Numerical values were calculated as means ± SD. For multiple sets of data, one-way ANOVA were used for significance analysis, and different letters such as a, b, and c indicate significant differences (*p* < 0.05). The comparison of the two sets of data used Student’s *t*-test followed by Duncan’s multiple range test in the SPSS statistics 17 (SPSS Inc., Chicago, IL, USA).

## 5. Conclusions

PalERF2 directly modulated the expressions of phosphorus starvation-responsive genes *PalPHL2* and *PalPHT1;4* to enhance the phosphorus starvation resistance and regulate drought response in poplar by binding to DRE motifs on the promoters of drought-responsive genes *PalRD20* and *PalSAG113*. Under drought stress, poplar recruits PalERF2 to elevate its phosphorus uptake capacity.

## Data Availability

The data that support the findings of this study are available from the corresponding author upon reasonable request.

## Supplementary Materials

The following supporting information can be downloaded at: https://www.mdpi.com/article/10.3390/ijms23095241/s1.
