# Supplementary material for: One AP2/ERF Transcription Factor Positively Regulates Pi Uptake and Drought Tolerance in Poplar
Source: Int J Mol Sci. 2022 May 8;23(9):5241. doi: 10.3390/ijms23095241 (PMC9099566; doi:10.3390/ijms23095241)
Supplement: Supplementary file 1 [file ijms-23-05241-s001.zip › Supplementary Figures.pdf]

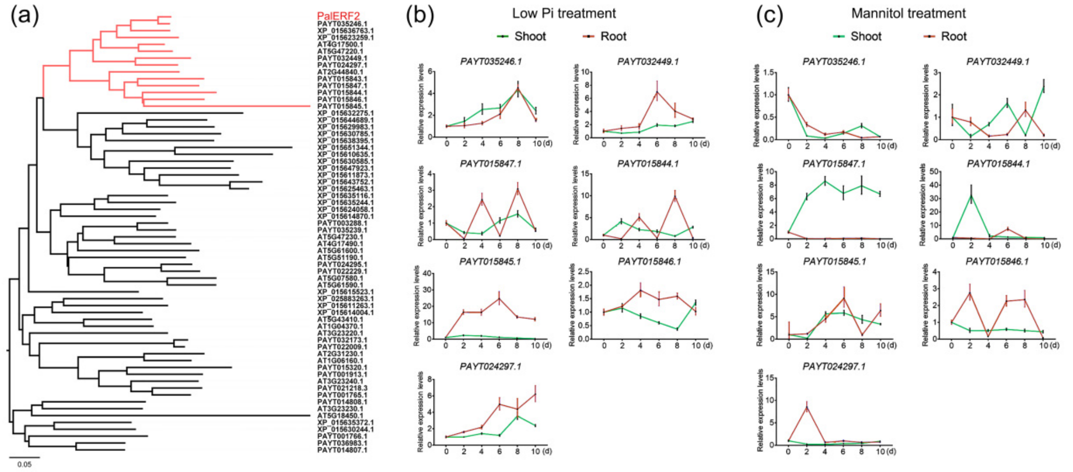

Figure S1. Phylogenetic analysis of B3 cluster of ERF subfamily members from Arabidopsis, *P. alba* var. *pyramidalis* and rice, and expression analysis of *PalERF2* and the paralogs in response to drought stress and low Pi condition. (A) 17 Arabidopsis, 23 poplar and 24 rice proteins from B3 cluster of ERF subfamily were aligned and constructed a neighbor-joining tree by MEGA6. The bootstrap value is 1000. The scale bar represents substitution rate per site. (B) The qRT-PCR analysis of *PalERF2* and its paralogs expression pattern in shoot and root of poplars under low Pi condition at the different times of treatment, respectively. (C) Analysis the expression level of *PalERF2* and its paralogs in shoot and root of poplar at different times after 150 mM mannitol treatment by qRT-PCR. Error bars indicate SD values from three biological replicates.

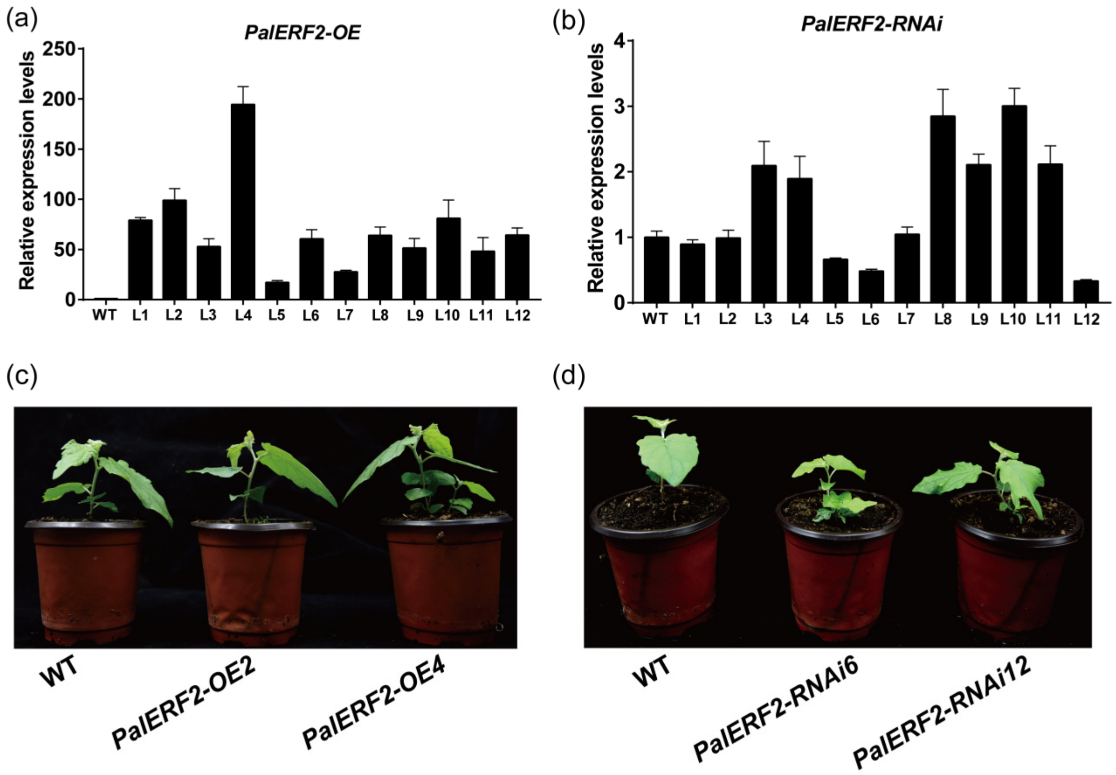

Figure S2. Identification of overexpression and RNAi lines of *PalERF2* in poplar. (A) The expression levels of overexpressing *PalERF2* poplars were determined by qRT-PCR. (B) The expression levels of PalERF2-RNAi lines were determined by qRT-PCR. (C) The cuttings of *PalERF2-OE* poplars (*PalERF2-OE2* and *PalERF2-OE4*) with the highest expression levels of *PalERF2*. (D) The cuttings of *PalERF2-RNAi* poplars (*PalERF2-RNAi6* and *PalERF2-RNAi12*) with the lowest expression levels of *PalERF2*.

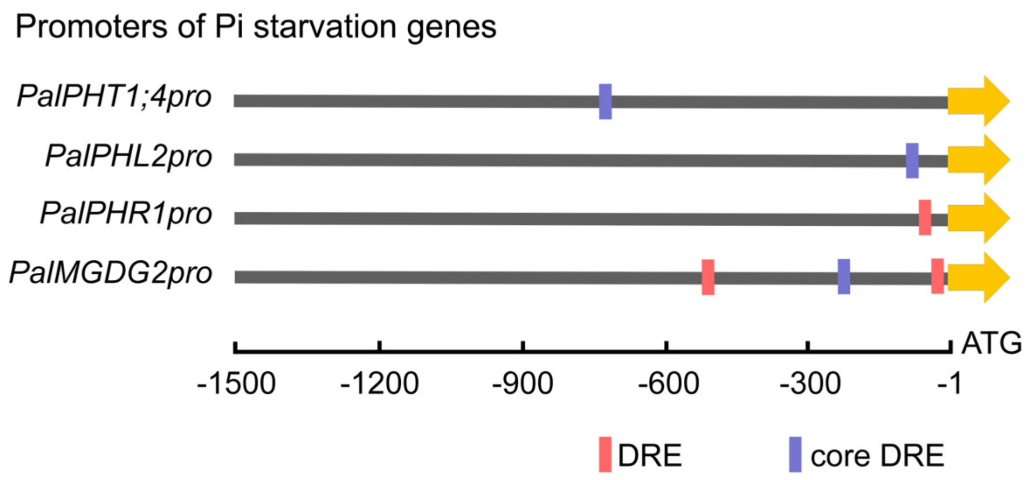

Figure S3. The distribution of DRE element in the promoters of the Pi starvation response PSR genes. *PalPHT1;4*, *PalPHL2*, *PalPHR1* have only one DRE element and *PalMGDG2* has three DREs on their promoters, respectively.

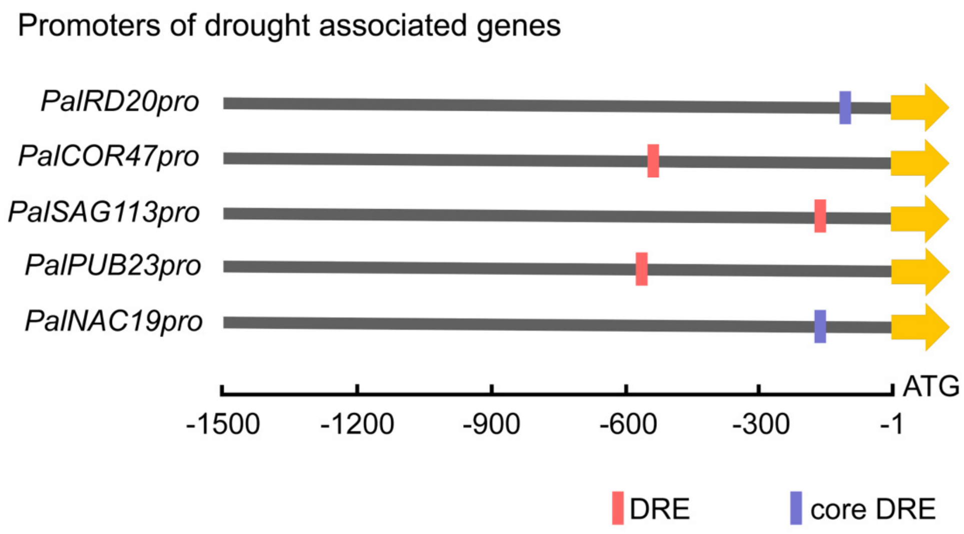

Figure S4. The distribution of DRE element in the promoters of drought responsive genes. *PalRD20*, *PalCOR47*, *PalSAG113*, *PalPUB23* and *PalNAC19* have only one DRE on their promoters, respectively.
